# Pharmacological Characterization of the Microsomal Prostaglandin E_2_ Synthase-1 Inhibitor AF3485 *In Vitro* and *In Vivo*

**DOI:** 10.3389/fphar.2020.00374

**Published:** 2020-04-02

**Authors:** Luigia Di Francesco, Annalisa Bruno, Emanuela Ricciotti, Stefania Tacconelli, Melania Dovizio, Paloma Guillem-Llobat, Maria Alessandra Alisi, Beatrice Garrone, Isabella Coletta, Giorgina Mangano, Claudio Milanese, Garret A. FitzGerald, Paola Patrignani

**Affiliations:** ^1^Department of Neuroscience, Imaging and Clinical Sciences, and Center for Advanced Studies and Technology (CAST), School of Medicine, G. d'Annunzio University, Chieti, Italy; ^2^Department of Systems Pharmacology and Translational Therapeutics, University of Pennsylvania, Philadelphia, PA, United States; ^3^Angelini Pharma S.p.A., Rome, Italy

**Keywords:** microsomal prostaglandin E_2_ synthase-1 inhibitors, microsomal prostaglandin E_2_ synthase-1, cyclooxygenases-2, whole blood, prostaglandin E_2_, TXB_2_, prostacyclin

## Abstract

**Rationale:**

The development of inhibitors of microsomal prostaglandin (PG)E_2_ synthase-1 (mPGES-1) was driven by the promise of attaining antiinflammatory agents with a safe cardiovascular profile because of the possible diversion of the accumulated substrate, PGH_2_, towards prostacyclin (PGI_2_).

**Objectives:**

We studied the effect of the human mPGES-1 inhibitor, AF3485 (a benzamide derivative) on prostanoid biosynthesis in human whole blood *in vitro*. To characterize possible off-target effects of the compound, we evaluated: i)the impact of its administration on the systemic biosynthesis of prostanoids in a model of complete Freund's adjuvant (CFA)-induced monoarthritis in rats; ii) the effects on cyclooxygenase (COX)-2 expression and the biosynthesis of prostanoids in human monocytes and human umbilical vein endothelial cells (HUVECs) *in vitro*.

**Methods:**

Prostanoids were assessed in different cellular models by immunoassays. The effect of the administration of AF3485 (30 and 100 mg/kg,i.p.) or celecoxib (20mg/kg, i.p.), for 3 days, on the urinary levels of enzymatic metabolites of prostanoids, PGE-M, PGI-M, and TX-M were assessed by LC-MS.

**Results:**

In LPS-stimulated whole blood, AF3485 inhibited PGE_2_ biosynthesis, in a concentration-dependent fashion. At 100μM, PGE_2_ levels were reduced by 66.06 ± 3.30%, associated with a lower extent of TXB_2_ inhibition (40.56 ± 5.77%). AF3485 administration to CFA-treated rats significantly reduced PGE-M (P < 0.01) and TX-M (P < 0.05) similar to the selective COX-2 inhibitor, celecoxib. In contrast, AF3485 induced a significant (P < 0.05) increase of urinary PGI-M while it was reduced by celecoxib. In LPS-stimulated human monocytes, AF3485 inhibited PGE_2_ biosynthesis with an IC_50_ value of 3.03 µM (95% CI:0.5–8.75). At 1μM, AF3485 enhanced TXB_2_ while at higher concentrations, the drug caused a concentration-dependent inhibition of TXB_2_. At 100 μM, maximal inhibition of the two prostanoids was associated with the downregulation of COX-2 protein by 86%. These effects did not involve AMPK pathway activation, IkB stabilization, or PPARγ activation. In HUVEC, AF3485 at 100 μM caused a significant (P < 0.05) induction of COX-2 protein associated with enhanced PGI_2_ production. These effects were reversed by the PPARγ antagonist GW9662.

**Conclusions:**

The inhibitor of human mPGES-1 AF3485 is a novel antiinflammatory compound which can also modulate COX-2 induction by inflammatory stimuli. The compound also induces endothelial COX-2-dependent PGI_2_ production *via* PPARγ activation, both *in vitro* and *in vivo*, which might translate into a protective effect for the cardiovascular system.

## Introduction

Prostanoids are inflammatory mediators produced from arachidonic acid (AA) metabolism (Patrignani and Patrono, 2015). The primary limiting step in this pathway is catalyzed by cyclooxygenases (COX), COX-1, and COX-2. COX-2 is induced under inflammatory conditions. The product of COX-isozyme activity is prostaglandin (PG) H_2_ which is then transformed to the different prostanoids *via* the action of the terminal synthases (Tilley et al., 2001; Simmons et al., 2004; Patrignani et al., 2005; Kang et al., 2007). Traditional nonsteroidal antiinflammatory drugs (tNSAIDs), such as ibuprofen, act by inhibiting both COX isozymes, and they have been proven to be effective in the reduction of pain and inflammation (Patrignani et al., 2005). However, these drugs are associated with an enhanced risk of severe gastrointestinal (GI) side-effects (Mitchell and Warner, 2006; Roth, 2011; Takeuchi, 2012) mainly due to the inhibition of COX-1 (Patrignani and Patrono, 2015).

Another group of NSAIDs is named coxibs (i.e., the selective COX-2 inhibitors). They have been developed to reduce the GI toxicity of tNSAIDs (Patrignani and Patrono, 2015). However, concerns regarding the safety profile of tNSAIDs and coxibs have risen due to their cardiovascular side-effects. The most plausible mechanism involves the reduction of the biosynthesis of vascular COX-2-dependent prostacyclin (PGI_2_), leaving unconstrained the COX-1-dependent production of thromboxane(TX)A_2_ from platelets together with other similar mediators (Sciulli et al., 2005; Grosser et al., 2006; Garcia Rodriguez et al., 2008).

A promising strategy to overcome this effect is through the inhibition of the terminal synthase involved in PGE_2_ biosynthesis, i.e., microsomal prostaglandin E_2_ synthase-1 (mPGES-1). mPGES-1 is a member of the membrane-associated proteins involved in eicosanoid and glutathione metabolism (MAPEG) superfamily. It is the major PGE synthase involved in PGE_2_ production during inflammation (Kudo and Murakami, 2005). It is considered a pharmacological target to achieve analgesia and antiinflammatory effects. MPGES-1 expression and activity is involved in PGE_2_ biosynthesis which is a key mediator of inflammation, pain, angiogenesis, fever, and tumorigenesis (Kamei et al., 2003; Trebino et al., 2003; Kamei et al., 2004; Saha et al., 2005; Chang and Meuillet, 2011).

Trebino et al. (2003) showed that deficiency of mPGES-1 gene in mice reduces antigen-induced inflammation, both in a model of delayed type hypersensitivity and in a model of arthritis. Furthermore, it was found a decrease in writhing, an indicator of inflammatory pain, that was indistinguishable in magnitude from that observed in mice treated with the tNSAID piroxicam (Trebino et al., 2003). These results suggest that mPGES-1 represents a target for the treatment of inflammatory diseases, such as arthritis.

PGE_2_ facilitates the neointimal hyperplasia response to injury through the activation of the PGE_2_ receptor subtype EP3α/β (Zhang et al., 2013). MPGES-1-derived PGE_2_ has been shown to contribute to vascular remodeling, stiffness, and endothelial dysfunction in hypertension likely through an increase of oxidative stress produced by NADPH oxidase and mitocondria (Avendaño et al., 2018).

This novel class of analgesic and antiinflammatory drugs may show an improved safety profile because mPGES-1 inhibitors can potentially elude the cardiovascular risk associated with coxibs and tNSAIDs by sparing vascular COX-2-dependent PGI_2_ biosynthesis (Samuelsson et al., 2007; Wang et al., 2008). PGI_2_ is a platelet antiaggregant and vasodilator, which antagonizes the platelet aggregating and vasoconstrictor actions of TXA_2_ and other similar stimuli (FitzGerald et al., 2001). Furthermore, PGI_2_ has antioxidant properties at vascular level (Egan et al., 2004; Di Francesco et al., 2009).

Studies with knockout (KO) mice for the PGI^2^ receptor (IP) have shown the role of this signaling pathway in the pathogenesis of inflammatory arthritis (Stitham et al., 2011). Thus, enhanced PGI_2_ biosynthesis associated with mPGES-1 inhibition might mitigate the consequence of PGE_2_ reduction. However, the finding of comparable efficacy of mPGES-1 deletion versus NSAID administration in mice (Trebino et al., 2003), which causes a concomitant reduction of the systemic biosynthesis of the two prostanoids, indirectly suggests a dominant role of PGE_2_ in this setting. Only a direct comparison of mPGES-1 inhibitors (once available to humans) versus tNSAIDs or coxibs will allow clarifying the possible contribution of enhanced PGI_2_ in arthritis.

A limitation in the development of mPGES-1 inhibitors is because the human enzyme has some differences in the aminoacid sequence versus the murine enzyme (Chang and Meuillet, 2011). Thus, the compounds intended for human application cannot be tested in animal models of pain and inflammation. Major challenges for drug development have also been the high plasma protein binding of lead structures (Koeberle et al., 2016). Moreover, it is necessary to clarify the possibility of the redirection of PGH_2_ substrate, leading to an increase in biosynthesis of PGI_2_ and PGD_2_, perhaps ameliorating the cardiovascular profile (Wang et al., 2006; Wang et al., 2008), but possibly worsening the efficacy (Stitham et al., 2011). However, attention should also be paid to the possible shift of PGH_2_ metabolism to TX-synthase and enhanced production of TXA_2_, a mediator of vasoconstriction and platelet activation (Boulet et al., 2004; Trebino et al., 2005; Cheng et al., 2006). Finally, the influence of selective mPGES-1 inhibition on the biosynthesis of other prostanoids may be cell-specific and should be evaluated both in *in vitro* and in *in vivo* systems, before a novel compound enters in the clinical development.

Several inhibitors of human mPGES-1 fail to potently inhibit murine enzyme isoforms, due to three individual amino acids, not conserved between human and murine mPGES-1 and located close to the active site of mPGES-1 (Korotkova and Jakobsson, 2014). However, despite this, they can cause antiinflammatory effects in rodent animal models (Koeberle et al., 2010), suggesting that they might have some off-target effects when administered *in vivo* in mice. We previously demonstrated that AF3442, a benzamide derivative belonging to the carbazole class of compounds, is a mPGES-1 inhibitor which reduced PGE_2_ generation both in isolated human monocytes and whole blood, i.e., in the presence of plasma proteins. In human monocytes inhibition of mPGES-1 did not translate into redirection of PGH_2_ metabolism towards other terminal PG synthases and at a high concentration of 100 μM, i.e., 100-fold higher than that causing a maximal inhibition of mPGES-1, AF3442 significantly reduced TXB_2_ biosynthesis and this effect was associated with an off-target impact on COX-2 expression (Bruno et al., 2010).

In this study we aimed: i) to characterize the effect of the human mPGES-1 inhibitor AF3485(a benzamide derivative) (Psarra et al., 2017) on prostanoid biosynthesis in human whole blood *in vitro;* ii) to verify *in vivo* the possible off-target effects of AF3485 by assessing its impact on systemic prostanoid biosynthesis in a rat model of complete Freund's adjuvant(CFA)-induced monoarthritis (Snekhalatha et al., 2013); iii) to evaluate *in vitro* the inhibitory effects of AF3485 on prostanoid biosynthesis and COX-2 expression in LPS-stimulated human monocytes, and endothelial cells.

## Materials and Methods

### Materials

The mPGES-1 inhibitor, AF3485 N-[9-(2-hydroxyethyl)-9H-carbazol-3yl]-2-(trifluoromethyl) benzamide (MW 398.38), was synthesized in Angelini S.p.A. (Rome, Italy). Lipopolysaccharide (LPS, derived from Escherichia Coli 026:B6), the irreversible peroxisome proliferator-activated receptor (PPAR)γ antagonist, GW9662, the complete Freund's adjuvant (CFA), the proteasome inhibitor MG132 (carbobenzoxy-Leu-Leu-leucinal), the cell-permeable AMP-activated protein kinase (AMPK) inhibitor, Compound C [ComC; {6-[4-(2-piperidin-1-ylethoxy) phenyl]-3-pyridin-4-ylpyrazolo [1,5-a] pyrimidine}], and cell-permeable activator of AMPK, AICAR (5-aminoimidazole-4-carboxamide-1-β-D-ribofuranoside) were purchased from Sigma-Aldrich (St. Louis, MO, USA). The selective COX-2 inhibitors, celecoxib was purchased from CCS.Chem.Co., Ltd (Zhejiang, China) while L-745337, was kindly provided by Merck Frosst Canada. The compounds were used in different experimental conditions, as described in detail below.

### Human Whole Blood Assays

Whole blood was drawn on different occasions from 3 healthy volunteers (age range: 30-35 years) who had not taken any NSAID during the 2 weeks preceding the study. This study was carried out following the recommendations of the Declaration of Helsinki after approval by the local Ethics Committee of “G. d'Annunzio” University of Chieti-Pescara, and informed consent was obtained from each subject. The inhibitory effect towards constitutive COX-1-dependent PGE_2_ production was assessed by evaluating PGE_2_ levels generated in whole blood allowed to clot for 60 minutes at 37°C. In clotting whole blood, PGE_2_ is mainly produced by platelets in response to endogenously generated thrombin through the activity of COX-1 (Patrono et al., 1980; Pratico and FitzGerald, 1996; Patrignani et al., 1999; Evangelista et al., 2006) and cPGES (cytosolic PGES) and/or mPGES-2. In fact, mPGES-1 is not detectable in platelets (Bruno et al., 2010). The parallel measurement of TXB_2_ (the stable hydrolysis product of TXA_2_, which is the primary product of endogenous AA metabolism in platelets) was performed to verify a possible off-target effect of AF3485 on platelet COX-1. The effects of AF3485 on inducible PGE_2_ generation were studied by assessing the levels of PGE_2_ produced in heparinized human whole blood stimulated for 24 h with LPS (10 µg/ml), a stimulus for the induction of both COX-2 and mPGES-1 in monocytes, in the presence of aspirin 10 µg/ml, added at time 0 to inhibit selectively the biosynthesis of platelet-derived prostanoids throughout the 24 h of incubation (Patrignani et al., 1994). The simultaneous measurement of TXB_2_ was performed to verify the impact of AF3485 on COX-2 and the possible redirection of the accumulated PGH_2_ substrate towards TXA_2_ synthase. In some experiments, L-745,337, a highly selective COX-2 inhibitor (Panara et al., 1995) was used. Increasing concentrations (0.01-100 μM) of AF3485 or DMSO vehicle were incubated with whole blood samples and then allowed to clot at 37°C for 1 h or with heparinized whole blood samples in the presence of LPS (10 μg/ml) at 37°C for 24 h. In some experiments, LPS-stimulated whole blood was incubated with ComC (10 µM) or AICAR (1mM), an AMPK inhibitor or activator, respectively. At the end of all experiments, prostanoids were measured in serum or plasma by previously described and validated immunoassays (Patrono et al., 1980; Patrignani et al., 1994).

### Isolated Human Monocytes

Human monocytes were separated from buffy coats (obtained from the blood bank of SS Annunziata Hospital, Chieti, Italy) by Ficoll-Paque (GE Healthcare Life Sciences, Bucks, UK), as previously described (Patrignani et al., 1994). Monocytes (1.5–2x10^6^ cells/ml) cultured in RPMI-1640 supplemented with 0.5% (v/v) of fetal bovine serum (FBS), 1% (v/v) penicillin/streptomycin and L-glutamine (2mM) (Sigma-Aldrich), were incubated with LPS (10 µg/ml ) at 37°C for 24 h in the presence of vehicle (DMSO) or increasing concentrations (0.01-100 µM) of the mPGES-1 inhibitor AF3485, in association or not with the PPARγ antagonist GW9662 (50 µM).PGE_2_ and TXB_2_ levels were measured in cell culture media by previously described and validated immunoassay techniques (Patrono et al., 1980; Patrignani et al., 1994), while COX-2, PPAR-γ and β-actin protein levels were evaluated in cell lysates by previously described Western blot techniques (Bruno et al., 2010).

### Monocytic Cell Line THP1

The THP1 cell line was obtained from the American Type Culture Collection (LGC Promochem, Milan, Italy) and cultured in RPMI 1640 containing 10% FBS, 1% penicillin/streptomycin, and 2 mM L-glutamine. Before every experiment, 1×10^6^ cells were cultured in 2 ml RPMI 1640 supplemented with 0.5% FBS for 16 h and then stimulated with 10 μg/ml LPS for 1 h in the presence of vehicle (DMSO) or MG-132 (10 µM) or AF3485 (100 µM). The expression levels of IκB-α and β-actin were evaluated in cell lysates by previously described Western blot techniques (Ricciotti et al., 2010).

### Endothelial Cell Cultures

Human umbilical vein endothelial cell (HUVEC), isolated from normal-term umbilical cords (collected at the Obstetrics and Gynaecological Unit, Padua University Hospital, Italy), as previously described (Trevisi et al., 2006), were grown and used for experiments at passage 2 or 3 (Di Francesco et al., 2009). This study conforms to the principles outlined in the Declaration of Helsinki for the use of human tissue. HUVEC (1x10^6^ cells) were seeded on gelatin covered 6-multiwell plates and grown for 4–5 days to form confluent monolayer in a humidified atmosphere of 95% air, 5% CO_2_, at 37°C in DMEM-medium 199 (50% vol/vol) supplemented with 15% FBS, 1% penicillin/streptomycin, 1% glutamine, 50 μg/ml endothelial cell growth supplement (ECGF) (RELIATech GmbH, Wolfenbuttel, Germany) and 100 U/ml heparin. The day before the experiment, the culture medium was changed with a DMEM-medium199 (50%, vol/vol), supplemented with 5% FBS, 1% glutamine, and antibiotics and treated with increasing concentration of AF3485 (0.1–100 µM) or vehicle DMSO for 24 h, in the absence or the presence of GW9662 (1–50 µM). In the conditioned medium 6-keto PGF_1α_ (the stable hydrolysis product of PGI_2_) levels were measured by radioimmunoassay as previously described (Patrignani et al., 1994). COX-2, PPAR-γ, and β-actin protein levels were evaluated in cell lysates by previously described Western blot techniques (Di Francesco et al., 2009).

### Animal Studies

Experiments were performed using male Sprague-Dawley rats weighing 200–250 g. The rats were housed in suspended wire mesh cages in a room maintained on a 12 h light/dark cycles (lights on at 07:00 h) and at 22°C–25°C with free access to food and water. The experiments were carried out in accordance with the guidelines established by the European Communities Council (Directive 2010/63/EU) and approved by the National Council on Animal Care of the Italian Ministry of Health. All efforts were made to minimize animal suffering and to use the minimal number of animals required to produce reliable results.

### CFA Injection and Pharmacological Treatment *In Vivo*

The rats were injected with 0.2 ml of CFA mixed with phosphate buffer saline in the ratio 1:1 into the right hind paw. For the pharmacological treatment, the rats were divided in four groups of 20 animals and chronically treated for 3 days (once a day), starting from CFA injection with: i) vehicle (0.5% methylcellulose solution); ii) AF3485 30mg/kg, intraperitoneal (i.p.); iii) AF3485 100 mg/kg, i.p.; iv) celecoxib 20 mg/kg, i.p. Twenty-four hour urine collections were performed after the last treatment. The levels of the major enzymatic metabolite of PGE_2_, i.e., 11a-hydroxy-9, 15-dioxo-2,3,4,5-tetranor-prostane-1,20-dioic acid (PGEM), of TXA_2_, i.e., 2,3-dinor-TXB_2_ (TXM), of PGI_2_, i.e., 2,3-dinor-6-keto- PGF_1α_ (PGIM) were assessed by liquid chromatography-mass spectrometry (LC-MS/MS), as previously described (Song et al., 2007).

### Western Blot Analysis

Cell lysate samples were loaded (20–50 µg/lane) onto SDS-PAGE and transferred to polyvinylidene fluoride (PVDF) membrane (Bio-Rad Laboratories, Hercules, CA). Membranes were saturated with a solution of 5% non-fat milk in tris-buffered saline-0.1% Tween-20 (TBS-Tween-20), and then incubated with anti-COX-2 (kindly provided by Merck Frosst, Canada), anti-PPAR-γ (Cayman Chemical), anti-IkB-α, or anti-β-actin (Santa Cruz Biotechnology, USA) polyclonal antibodies for 1 h at room temperature (Ricciotti et al., 2010). Then, the membranes were washed in TBS-Tween-20 and incubated with the secondary antibodies. Finally, the membranes were washed in TBS-Tween-20, and all blots were developed by using ECL plus detection according to the manufacturer's instructions (GE Healthcare Life Sciences, Bucks, UK).

### Statistical Analysis

Results are expressed as mean ± SEM, unless otherwise stated. For the experiments of LPS-stimulated monocytes and whole blood, the production of PGE_2_ and TXB_2_ was subtracted from the levels of the prostanoids measured in the presence of vehicle (DMSO). Statistical analysis was performed with Student's t-test or one-way ANOVA and Newman-Keuls multiple comparison test (using PRISM, GraphPad, San Diego, CA). Values of P < 0.05 were considered statistically significant. Concentration-response curves were fitted (using PRISM, GraphPad, San Diego, CA), and IC_50_ (concentration of the compound to inhibit by 50% prostanoid production) values were reported.

## Results

### Effect of AF3485 on Prostanoid Production in LPS-Stimulated Human Whole Blood

AF3485 is a benzamide derivate developed by the Angelini S.p.A. It inhibits human, but not mouse, recombinant mPGES-1 expressed in bacterial membranes [IC_50_: 2.55µM] and selectively restrains IL-1β-induced PGE_2_ production in A549 cells [IC_50_: 1.98µM] (Finetti et al., 2012). An important issue to address in the development of mPGES-1 inhibitors is related to their ability to inhibit PGE_2_ in the presence of plasma proteins (Koeberle et al., 2010). In this regard, we assessed the capacity of AF3485 to inhibit PGE_2_ production in LPS-stimulated human whole blood (Patrignani et al., 1994).

**Figure 1:**
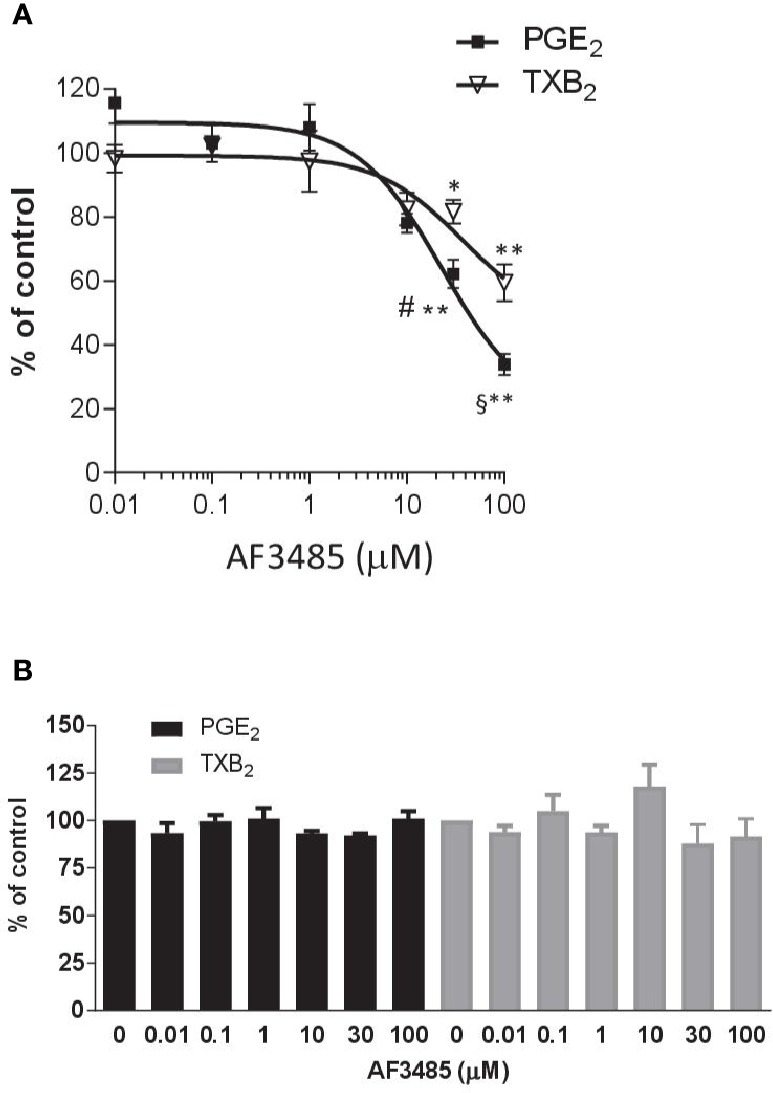
Effects of AF3485 on prostanoid production in human LPS- or thrombin-stimulated whole blood. **(A)** Increasing concentrations of AF3485 (0.01–100 µM) or DMSO vehicle were incubated with 1 ml of heparinized human whole blood stimulated with LPS (10 µg/ml) for 24 h. PGE_2_ and TXB_2_ were assessed by immunoassays. Results were reported as % of control (DMSO vehicle) (mean ± SEM, n = 4-5). **P < 0.01, *P < 0.05 versus vehicle (DMSO). ^#^P < 0.05, ^§^P < 0.01, PGE_2_ versus TXB_2_ (Student's t-test). **(B)** AF3485 (0.01 µM–100 µM) or DMSO vehicle was incubated with 1 ml of whole blood samples that were allowed to clot for 60 min at 37°C. PGE_2_ and TXB_2_ levels were measured. Results are reported as % of control (DMSO vehicle) (mean ± SEM, n = 3-4)(One-way ANOVA). LPS, Lipopolysaccharide; PGE_2_, prostaglandin E_2._

AF3485 caused a concentration-dependent reduction in PGE_2_ production (**Figure 1A**). At 30 µM, the compound caused inhibition of 37.78 ± 4.45% while at 100 µM, it was 66.06 ± 3.30%. In LPS-stimulated whole blood, TXB_2_ is also produced by induced COX-2 in LPS stimulated monocytes. In fact, the highly selective COX-2 inhibitor L-745,337 (Panara et al., 1995) caused a comparable concentration-dependent inhibition of PGE_2_ and TXB_2_ (**Supplementary Figure 1**). TXB_2_ levels were measured to determine if the inhibition of PGE_2_ biosynthesis by AF3485 could lead to a redirection of PGH_2_ to TXA_2_ synthase. TXB_2_ levels detected in LPS-stimulated whole blood were comparable to those of PGE_2_ (**Supplementary Table 1**). As shown in **Figure 1A**, the compound caused a concentration-dependent reduction of TXB_2_ levels. AF3485, at 100 µM, caused a significant (P < 0.01) inhibition in the production of TXB_2_ by 40.56 ± 5.77%. At 30 and 100 µM, the inhibition of PGE_2_ generation was significantly greater than that of TXB_2_. These results show that in the presence of plasma proteins, AF3485 affects mPGES-1 activity, but high concentrations are required to cause a clinically relevant inhibition of the proinflammatory PGE_2_ in LPS-stimulated whole blood. However, at these concentrations, the compound may also affect COX-2-dependent TXB_2_ in whole blood stimulated with LPS. These results may suggest that the compound also interferes with the induction of COX-2 in response to LPS.

Next, we assessed the impact of AF3485 on prostanoid biosynthesis in thrombin-stimulated whole blood (serum); in this assay, prostanoids are generated from platelet COX-1 and constitutive downstream synthases. In platelets, COX-2 and mPGES-1 are not detectable (Bruno et al., 2010). As reported in **Figure 1B**, none of the tested concentrations of AF3485 (0.01 µM to 100 µM) caused significant changes in the levels of thrombin-induced PGE_2_ and TXB_2_, showing that the compound does not affect COX-1, TXA_2_ synthase, and the other PGE_2_ synthases.

### Effect of AF3485 Administration on the Systemic Biosynthesis of CFA-Induced Prostanoids *In Vivo*

**Figure 2:**
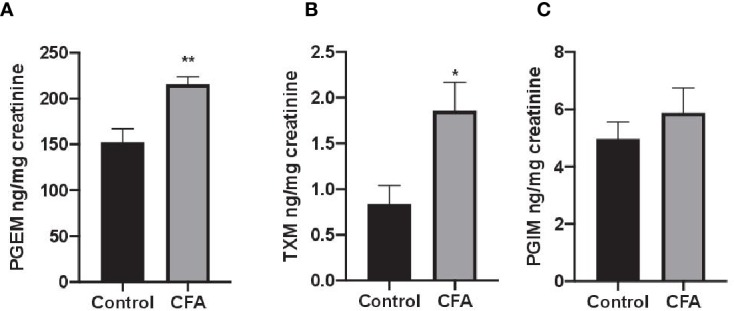
Effects of CFA injection on the systemic biosynthesis of prostanoids in rats. The systemic biosynthesis of PGE_2_, TXB_2_, and PGI_2_ was assessed by measuring their urinary enzymatic metabolites, i.e., PGEM **(A)**, TXM **(B)** and PGIM **(C)**, respectively, by LC-MS/MS. Data are reported as mean ± SEM (n=5–8). Metabolite levels were corrected for urinary creatinine and expressed as ng/mg creatinine. **P < 0.01, *P < 0.05 CFA injection versus control (Student's t-test). CFA, complete Freund's adjuvant; PGE_2_, prostaglandin E_2_.

To assess the impact AF3485 on prostanoid biosynthesis *in vivo*, we used a model of inflammation induced by CFA-injection in rats. The selective COX-2 inhibitor celecoxib was also tested in comparison. In this model, we measured the urinary enzymatic metabolites of PGE_2_, TXB_2_, and PGI_2_, i.e., PGEM, a marker of systemic PGE_2_ biosynthesis which can derive mainly, but not exclusively, from COX-2 in inflammatory cells; TXM, a marker of systemic TXB_2_ biosynthesis derived mainly (approximately 70%) from platelet COX-1 under basal conditions; PGIM, a marker of systemic PGI_2_ biosynthesis mostly from vascular COX-2 under basal conditions (FitzGerald et al., 1983; Song et al., 2007; Saul et al., 2019). As reported in **Figures 2A–C**, CFA injection caused a significant increase in the systemic biosynthesis of PGE_2_ (P < 0.05) and TXB_2_ (P < 0.01), while the levels of PGIM were unaffected.

**Figure 3:**
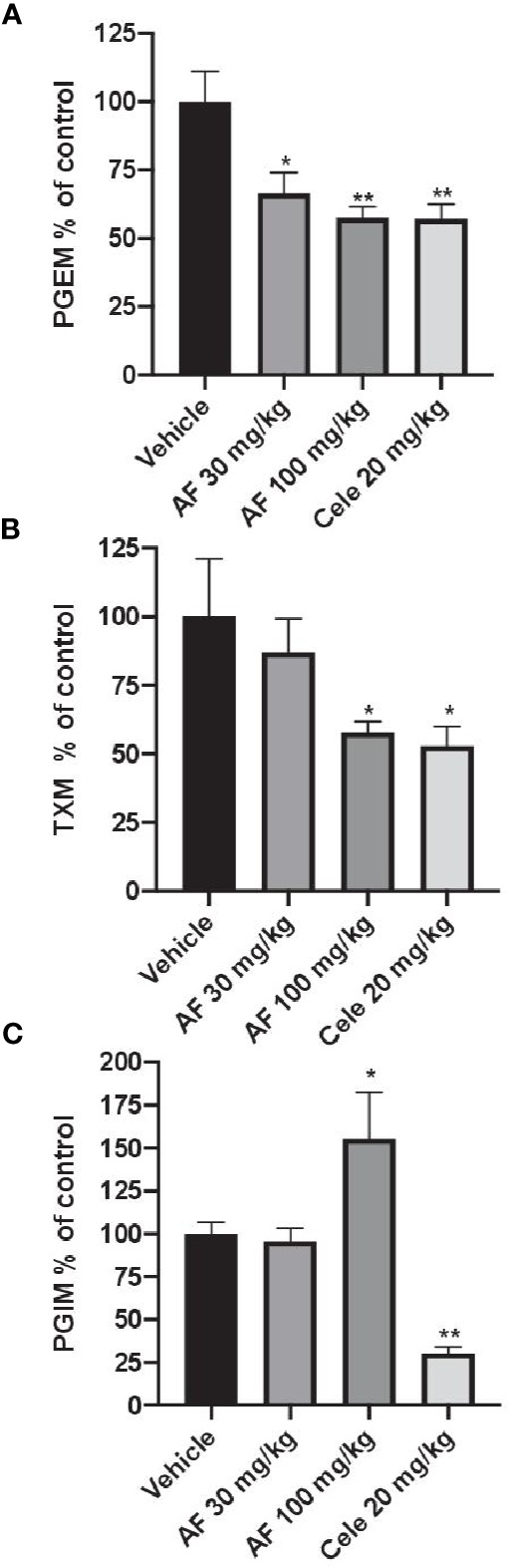
Effects of AF3485 on the systemic biosynthesis of prostanoids in CFA-injected rats. Animals were chronically treated for 3 days with: i) vehicle (0.5% methylcellulose solution); ii) AF3485 30mg/kg, intraperitoneal (i.p.) (AF 30); iii) AF3485 100 mg/kg, i.p. (AF 100) and iv) celecoxib 20 mg/kg, i.p. (Cele 20). Urinary PGEM **(A)**, TXM **(B)**, and PGIM **(C)**, were assessed by LC-MS/MS, corrected for urinary creatinine, and reported as % of control (vehicle). Values are reported as mean ± SEM (n=13–20) *P < 0.05, **P < 0.01 versus vehicle (One-way ANOVA and Student's t-test). CFA, complete Freund's adjuvant.

In CFA-injected rats, the treatment with AF3485 both at 30 mg/kg and at 100 mg/kg produced a significant reduction in urinary PGEM levels (33.49 ± 7.60%, P < 0.05 vs vehicle and 42.45 ± 4.12%, P < 0.01 vs vehicle, respectively) which was similar in extent to that caused by celecoxib administration (20 mg/kg) (42.84 ± 5.27%) (**Figure 3A**). As shown in **Figure 3B**, the systemic biosynthesis of TXB_2_ was not significantly affected by 30 mg/kg of AF3485, while at 100 mg/kg, the compound reduced CFA-induced TXB_2_ biosynthesis *in vivo* similarly to celecoxib treatment (42.18 ± 4.02% and 47.12 ± 7.07%, respectively).

The systemic biosynthesis of PGI_2_ was significantly reduced by celecoxib (20 mg/kg), (69.70 ± 3.74%, P < 0.01 versus vehicle). AF3485, at 30 mg/kg, did not significantly affect the biosynthesis of PGI_2_. However, the administration of 100 mg/kg of the compound caused a significant increase in PGI_2_ biosynthesis (55.42 ± 27.22%, P < 0.05 versus vehicle) (**Figure 3C**).

Altogether these results led us to hypothesize that in CFA-induced arthritic rat model, AF3485 affects the COX-2 pathway in inflammatory cells, similarly to celecoxib. In vascular cells, AF3485 may enhance COX-2 dependent PGI_2_ biosynthesis, possibly by inducing COX-2 expression. To address this hypothesis, we assessed the impact of AF3485 *in vitro* on prostanoid production and COX-2 protein levels in monocytes and endothelial cells.

### Effect of AF3485 on Prostanoid Production and COX-2 Expression in LPS-Stimulated Monocytes

We have previously shown that in human monocytes, LPS causes a significant increase in TXB_2_, PGE_2_, and 6-keto-PGF_1α_ (the nonenzymatic hydrolysis product of PGI_2_, and a marginal product of AA metabolism) (Bruno et al., 2010).

**Figure 4:**
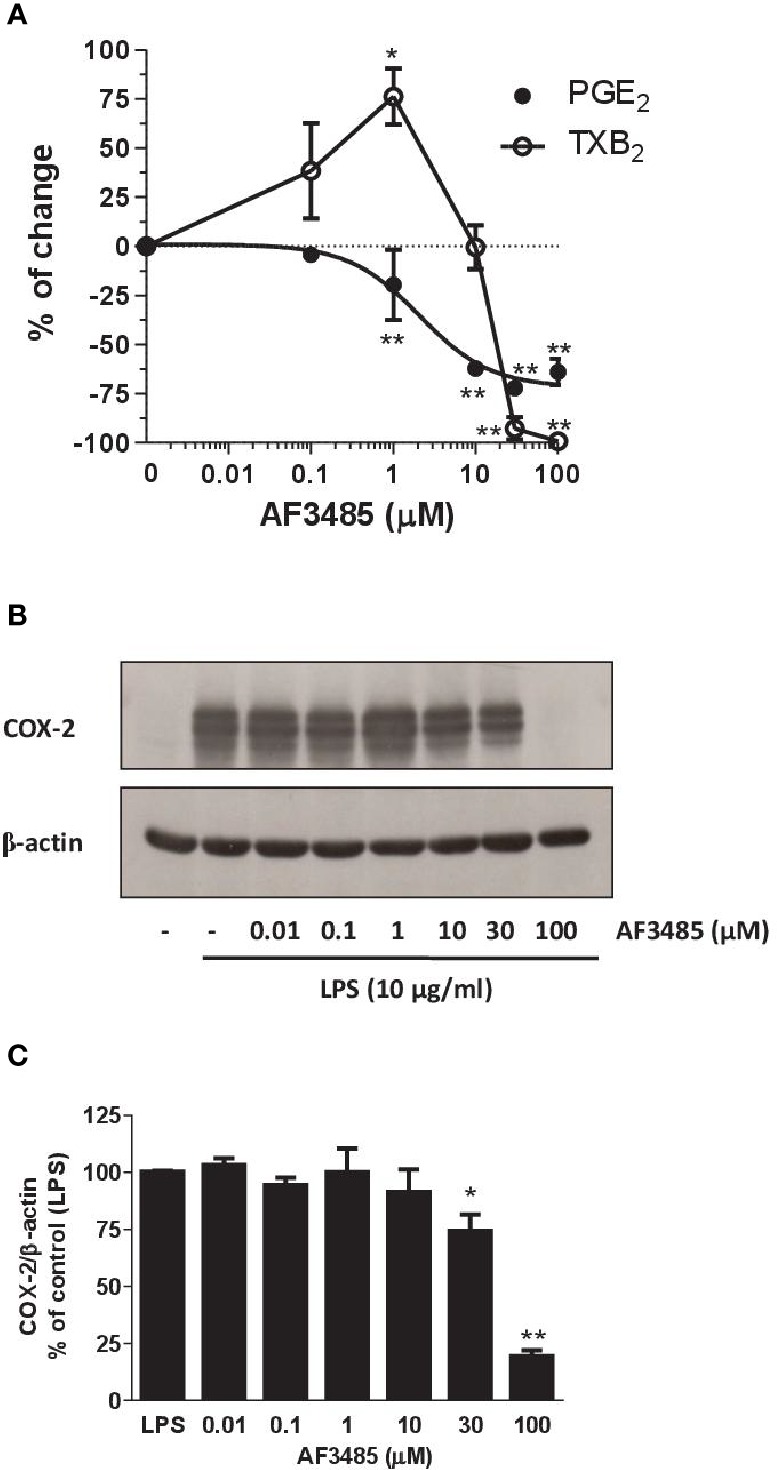
Effects of AF3485 on prostanoid biosynthesis and COX-2 expression in LPS-stimulated monocytes. Increasing concentrations of AF3485 (0.01–100 µM) or DMSO vehicle were added to isolated human monocytes (1.5–2 x 10^6^ cells/ml) in the presence of LPS (10 µg/ml) for 24 h at 37 °C. **(A)** PGE_2_ and TXB_2_ levels were measured in conditioned media by immunoassays. The data are reported as % of control (DMSO vehicle) (mean ± SEM, n=4–5). *P < 0.05, **P < 0.01 versus control (DMSO vehicle). (Student's t-test). **(B)** COX-2 was analyzed by Western blot; β-actin was used as protein loading control. The figure is representative of three different experiments. **(C)** Densitometric analysis of COX-2 expression; blots were normalized to β-actin and the data were reported as % of control (LPS), bars are mean ± SEM, n=3. *P < 0.05 and **P < 0.01 versus LPS (Student's t-test). PGE_2_, prostaglandin E_2._

As reported in **Figure 4A**, AF3485 treatment caused a concentration-dependent reduction of LPS-induced production of PGE_2_ in human monocytes with an IC_50_ value of 2.03 µM (95% CI: 0.5–8.75). AF3485 10 µM, caused an inhibitory effect on PGE_2_ production by 62.30 ± 3.14% (P < 0.01 vs DMSO vehicle), which was only marginally enhanced at higher concentrations. At 30 and 100 µM, the biosynthesis of PGE_2_ was reduced by 72.07 ± 1.63% and 63.93 ± 6.84%, respectively (**Figure 4A**).

At concentrations of AF3485 up to 1 µM, an increase in TXB_2_ levels (76.2 ± 14.3%, P< 0.05) associated with the reduction of PGE_2_ was detected (**Figure 4A**); at higher concentrations, the compound inhibited the production of TXB_2_ with an IC_50_ value of 22 µM (**Figure 4A**). At 30 and 100 µM, TXB_2_ biosynthesis was significantly inhibited by 92.70 ± 5.76% and 99.17 ± 0.83%, respectively (**Figure 4A**).

Next, we assessed whether AF3485 affected the expression of COX-2 in LPS-stimulated monocytes. As shown in **Figures 4B, C**, AF3485 up to 10 µM did not significantly affect the levels of COX-2 expression, while at 30 µM and 100 µM, the compound caused a significant (P < 0.01 versus control) reduction of COX-2 levels by 25.60 ± 7.16% and 80.53 ± 2.55%, respectively (assessed as ratio of their optical density normalized to the optical density of β-actin).

### AMPK Pathway and the Inhibitory Effect of AF3485 on PGE_2_ Production in LPS-Stimulated Whole Blood *In Vitro*

AMPK is an enzyme of cellular energy homeostasis as a metabolic stress-sensing protein (Steinberg and Kemp, 2009). Activation of AMPK and the inhibition of downstream protein p65 NF-κB attenuates inflammatory arthritis (Guma et al., 2015).

**Figure 5:**
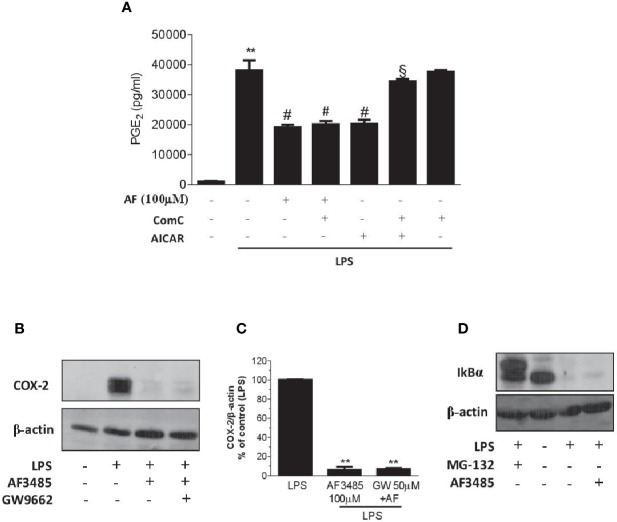
Effects of pharmacological modulation of AMPK, PPARγ or IkBα on the inhibition of PGE_2_ biosynthesis and COX-2 expression by AF3485 in human blood leukocytes. **(A)** AF3485 (100 µM) was incubated with 1 ml of heparinized human whole blood stimulated with LPS (10 µg/ml) for 24 h, in the presence or absence of ComC (10 µM) or AICAR (1 mM); plasma PGE_2_ was assessed by immunoassay; values are reported as mean ± SEM, n=3. **P < 0.01 versus vehicle; ^#^P < 0.01 versus LPS; ^§^P < 0.01 versus AICAR. (One-way ANOVA and Student's t-test). **(B)** LPS-stimulated monocytes (0.8-1x10^6^ cells) were treated with AF3485 (100 µM) or vehicle (DMSO), in the presence or absence of the PPARγ antagonist GW9662 (50 µM) for 24 h; COX-2 levels were assessed by Western blot and β-actin was used as protein loading control; a representative experiment (from three independent experiments) is shown. **(C)** Densitometric analysis of COX-2 expression normalized to β-actin; values are reported as % of control (LPS), mean ± SEM, n = 3. **P < 0.01 versus LPS (Student's t-test). **(D)** Levels of IκBα assessed by Western blot in THP-1 cells (1x10^6^ cells) stimulated with LPS (10 µg/ml) for 1 h in the absence or presence of AF3485 (100 µM) and the effect of the proteasome inhibitor MG-132 (10 µM) was evaluated; β-actin levels were used as protein loading control.

We assessed whether the reduction of PGE_2_ biosynthesis in human whole blood by AF3485 was dependent on the activation of AMPK. First, we tested the effect of the AMPK activator AICAR. As shown in **Figure 5A**, similarly to AF3485, AICAR reduced PGE_2_ production in LPS-stimulated whole blood. Then, we verified whether these effects were prevented by the AMPK inhibitor ComC (also called dorsomorphin). ComC reverted the inhibitory effect of AICAR on PGE_2_ production in human whole blood stimulated with LPS for 24 h (**Figure 5A**). In contrast, ComC did not affect the reduction of PGE_2_ production by AF3485. ComC did not change PGE_2_ biosynthesis in LPS-whole blood when incubated alone (**Figure 5A**). Altogether these results suggest that the off-target effect of AF3485 on PGE_2_ biosynthesis in whole blood inflammatory cells in response to LPS does not involve the activation of the AMPK pathway.

### PPARγ Activation and the Effect of AF3485 on Monocyte COX-2 Downregulation

PPARγ is a member of the nuclear hormone receptor superfamily of ligand-activated transcription factors that have been shown to regulate inflammatory responses and assist in the resolution of inflammation (Kawahito et al., 2000; Fahmi et al., 2002). In macrophage-like differentiated U937 cells, PPARγ activation has been reported to suppress COX-2 promoter activity by interfering with the NF-kB signaling pathway (Inoue et al., 2000). Thus, we explored whether AF3485 caused COX-2 downregulation *via* PPARγ activation in human monocytes. In isolated human monocytes incubated in the absence and the presence of LPS, PPARγ was expressed and AF3485 did not affect its protein levels (**Supplementary Figure 2**).

The use of the PPARγ antagonist GW9662 did not produce any significant change in the reduction of COX-2 expression by AF3485 (**Figures 5B, C**), thus excluding the role of PPARγ activation by AF3485 on the downregulation of COX-2 in LPS-activated monocytes.

### Effect of AF3485 on IkB Stability in the Monocytic Cell Line THP-1

LPS activates the transcription factor NF-kB through the interaction with toll-like receptor (TLR) 4. This leads to NF-kB binding to the target genes, including COX-2 (Tanabe and Tohnai, 2002). LPS triggers several cascades of intracellular signaling events, including those that lead to activation of the IkB kinase (IKK), which by phosphorylating IkB leads to ubiquitination and proteasomal degradation (Chen et al., 1995); then, NF-kB is released and moves to the nucleus and exerts transactivation (Oeckinghaus and Ghosh, 2009).

We explored whether AF3485 affects COX-2 induction in response to LPS *via* an effect on IκB degradation. In the monocytic cell line THP-1, AF3485 100 µM was unable to revert the LPS-dependent degradation of IκB (**Figure 5D**). In contrast, the treatment with the proteasome inhibitor, MG-132 (10 µM), was associated with the accumulation of IκB in the cells treated with LPS (**Figure 5D**). Thus, the interference of IκB degradation was not involved in AF3485-dependent downregulation of LPS-induced COX-2.

### Effects of AF3485 on PGI_2_ Biosynthesis and COX-2 Expression in HUVEC

**Figure 6:**
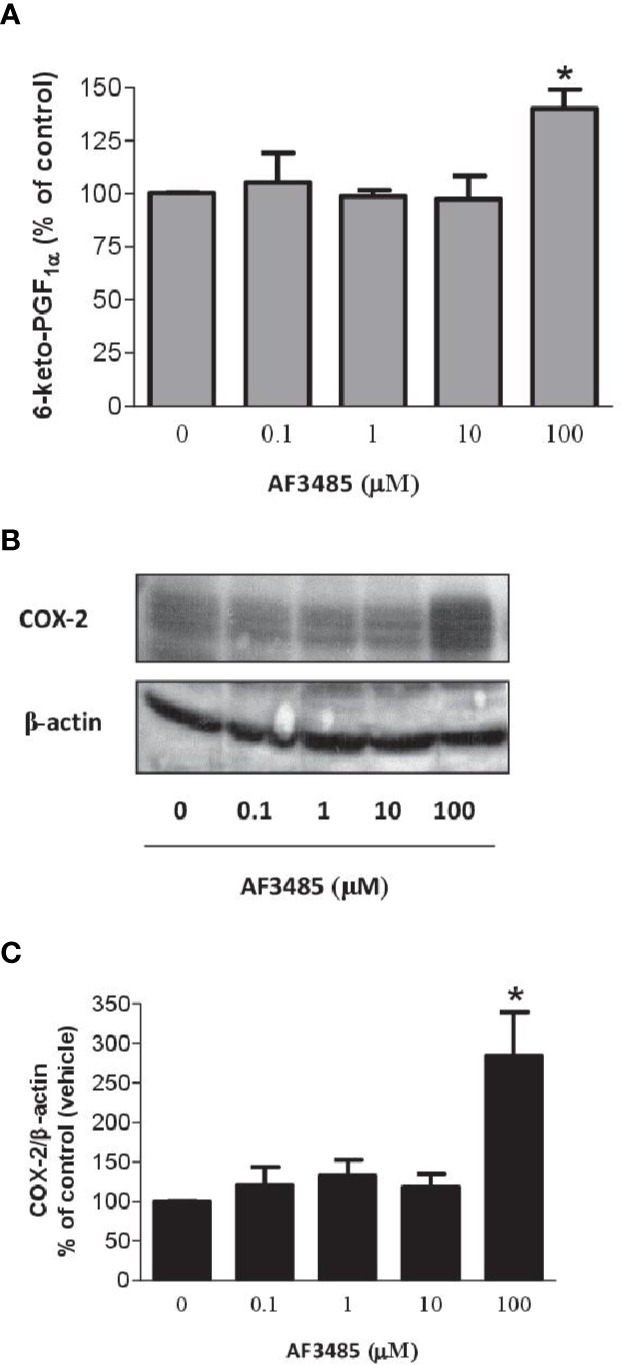
Effects of AF3485 on PGI_2_ biosynthesis and COX-2 expression in HUVEC. **(A)** HUVEC (1x10^6^ cells) were treated with increasing concentrations of AF3485 for 24 h and the conditioned medium was collected to assess the nonenzymatic hydrolysis product of PGI_2_, i.e., 6-keto-PGF_1α_;values are shown as mean ± SEM, n = 4; *P < 0.05 versus vehicle (DMSO). **(B)** Western blot analysis of COX-2 expression in HUVEC; β-actin levels were used as protein loading control; the blot is representative of three different experiments. **(C)** In HUVEC treated with AF3485, densitometric analysis of COX-2 bands normalized to β-actin was performed; results are reported as % of control (DMSO vehicle) (mean ± SEM, n = 3); *P < 0.05 versus control (DMSO) (Student's t-test).

COX-2 is the source of vascular PGI_2_ biosynthesis in humans, which exhibits properties of relevance to atheroprotection, inhibiting platelet activation, vascular smooth muscle contraction and proliferation, leukocyte-endothelial cell interactions, and cholesteryl ester hydrolase (Grosser et al., 2006). We studied the impact of AF3485 on PGI_2_ biosynthesis by assessing 6-keto-PGF_1α_ in HUVEC cultured with AF3485. The compound did not affect the release of 6-keto-PGF_1α_ up to 10 µM, while at 100 µM of AF3485, a significant increase of 6-keto-PGF_1α_ levels (P < 0.05 vs vehicle) (**Figure 6A**) was detected. This effect was associated with a significant (P < 0.05) induction of the protein levels of COX-2, assessed by Western blot (**Figures 6B, C**), by AF3485 (100 µM).

### PPARγ Activation and the Effect of AF3485 on PGI_2_ Biosynthesis and COX-2 Expression in HUVEC

**Figure 7:**
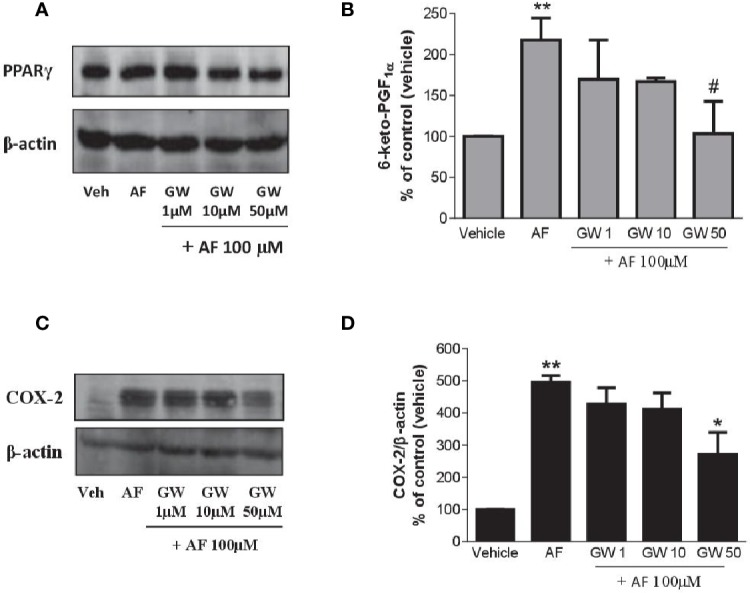
Effects of a PPARγ antagonist on the increase of PGI_2_ biosynthesis and COX-2 expression by AF3485 in HUVEC. HUVEC (1x10^6^ cells) were incubated with AF3485 (100 µM) alone or in combination with GW9662 (1–50 µM) for 24 h. **(A)** Western blot analysis of PPARγ is reported; β-actin levels were used as protein loading control. **(B)** Assessment of 6-keto-PGF_1α_ in the conditioned medium of HUVEC by immunoassay; data are reported as percentage of control (vehicle). **P < 0.01 versus vehicle; ^#^P < 0.05 versus AF3485 100 µM. **(C)** Western blot analysis of COX-2; β-actin levels were used as protein loading control. **(D)** Densitometric analysis of COX-2 expression normalized to β-actin levels; results are reported as % of control (DMSO vehicle) (mean ± SEM, n = 3); **P < 0.01 versus control (DMSO); *P< 0.05 versus AF3485 100 µM (One-way ANOVA and Student's t-test). COX, cyclooxygenases.

As shown in **Figure 7A**, HUVEC expressed PPARγ, and the levels were not affected by AF3485. The PPARγ antagonist GW9662 caused a concentration-dependent reduction of 6-keto-PGF_1α_ released in response to the exposure of HUVEC to AF3485 (**Figure 7B**). GW9662 (50 µM) almost wholly abolished the increase in 6-keto-PGF_1α_ caused by AF3485 (**Figure 7B**). This effect was associated with a significant (P < 0.05) reduction in the COX-2 expression that had been induced by AF3485 100 µM (**Figures 7C, D**).

Altogether these results suggest that in endothelial cells, AF3485 may induce COX-2 expression and PGI_2_ biosynthesis *via* PPARγ modulation.

## Discussion

MPGES-1 inhibitors are potential analgesic and antiinflammatory agents that are under clinical development (Chang and Meuillet, 2011; Korotkova and Jakobsson, 2014). They have the promise to restrain the cardiovascular toxicity of tNSAIDs and coxibs (Wang et al., 2008, 2011). Selective inhibition of mPGES-1 may redirect the accumulated PGH_2_ substrate to vascular PGI synthase; thus, increasing biosynthesis of PGI_2_, which plays a protective role for the cardiovascular system (Wang et al., 2006). However, enhancement of other prostanoids, including TXA_2_, might confound such a theoretical benefit.

The mPGES-1 inhibitor AF3485 is a benzamide derivative which was synthesized to affect human mPGES-1 (Finetti et al., 2012). The compound neither interacted with the mouse mPGES-1 enzyme nor affected its activity (Finetti et al., 2012). We used the whole blood assay to characterize the impact of AF3485 to inhibit the capacity of clinically relevant cells, such as platelet COX-1 and monocyte COX-2, to generate prostanoids (Patrono et al., 1980; Patrignani et al., 1994 and Patrignani and Patrono, 2015). The whole blood assay is important because it allows verifying the inhibitory effect on prostanoid biosynthesis in the presence of plasma proteins. A significant limitation in the development of mPGES-1 inhibitors is their high plasma protein binding (Koeberle et al., 2016).

We show that AF3485 inhibited human whole blood PGE_2_ generation in response to LPS, but high concentrations (100 µM) were requested to affect LPS-induced PGE_2_ biosynthesis in whole blood to a degree relevant to an analgesic and anti-inflammatory effects (i.e., approximately 70%) (Patrignani and Patrono, 2015). However, at this high concentration, the compound also affected COX-2-dependent TXB_2_ in LPS-stimulated whole blood and monocytes. In isolated monocytes, AF3485 was incubated with low concentrations of plasma proteins, i.e., 0.5% of FBS. The compound was more potent in this model than in whole blood. The most plausible explanation is the extensive binding to plasma proteins, which reduces the concentration of free AF3485 available to inhibit mPGES-1. In this experimental condition, we tested the possible redirection of PGH_2_ to TXA_2_. In isolated monocytes, AF3485 was more potent than in whole blood; thus, it was possible to separate the effect on mPGES-1 (i.e., the reduction of PGE_2_ production associated with enhanced TXA_2_ generation) from that on COX-2. AF3485 affected COX-2 at higher concentrations and prevented the accumulation of PGH_2_ and the increase of TXA_2_.

We show that AF3485 interferes with the induction of COX-2 in response to LPS. This effect did not involve AMPK pathway activation, IkB stabilization, or PPARγ activation. However, in contrast, the compound may induce COX-2 and PGI_2_ biosynthesis in endothelial cells at the basal state through the activation of PPARγ. These results suggest that AF3485 may influence PPARγ activity. AF3485 may induce COX-2 expression by PPAR-γ response elements (PPRE) in the 5'-regulatory region of COX-2 gene (Pontsler et al., 2002). The reduction of COX-2 expression by AF3485 in monocytes stimulated with LPS might involve the mechanism described by Pascual et al. (2005). They show a new model for trans-repression in which ligand-dependent SUMOylation of PPARγ results in its recruitment to the promoters of inflammatory genes where it inhibits transcription by preventing clearance of corepressor complexes. Further studies are requested to verify whether AF3485 can cause the SUMOylation of PPARγ in LPS-stimulated monocytes.

These off-target effects of AF3485 on the prostanoid pathway were found *in vivo* by the administration of AF3485 to rats in a model of CFA-induced monoarthritis (Snekhalatha et al., 2013). AF3485 is unable to affect murine mPGES-1. We assessed the impact on urinary levels of major enzymatic metabolites of PGE_2_, TXB_2_, and PGI_2_, i.e., PGEM, TXM, and PGIM, respectively. They are reliable and noninvasive indexes of the systemic biosynthesis of the parent prostanoids (FitzGerald et al., 1983; Song et al., 2007). The use of the selective COX-2 inhibitor celecoxib showed that enhanced urinary PGEM and TXM detected in response to the administration of CFA was dependent on COX-2 activity. Urinary PGIM was not enhanced in CFA-induced monoarthritis. PGIM is considered a marker of vascular COX-2-dependent PGI_2_ biosynthesis (McAdam et al., 1999). Celecoxib reduced the urinary levels of PGIM in CFA-treated rats. The administration of AF3485 at 100 mg/kg caused a comparable inhibitory effect of PGEM and TXM to celecoxib. Considering that AF3485 is not inhibiting rat mPGES-1, these effects show that also *in vivo*, the compound interferes with the biosynthesis of COX-2-dependent prostanoids induced by the inflammatory response to CFA administration.

An intriguing result was that *in vivo*, the compound caused an increase of the urinary levels of PGIM; PGIM is mainly derived by vascular COX-2 (Yu et al., 2012), and indeed we found that celecoxib profoundly reduced it. This finding prompted us to verify the hypothesis that AF3485 induced PGI_2_ production in endothelial cells associated with COX-2 induction. We used HUVEC only as a tool to assess the mechanism of COX-2 induction. As previously reported, HUVEC also produce PGE_2_ and PGF_2α_ (Di Francesco et al., 2009). They are cells of fetal origin that have a different pattern of prostanoid biosynthesis as compared to endothelial cells from the macrocirculation where PGI_2_ is the dominant prostanoid (Grosser et al., 2006). This is probably due to different expression of prostanoid synthases in different location of the vascular tree. We aimed only to confirm that in endothelial cells AF3485 can upregulate COX-2 and increase the generation of PGI_2_. Thus, we did not evaluate the other prostanoids.

In conclusion, the selective inhibitor of human mPGES-1 AF3485 is a novel antiinflammatory compound acting *via* the reduction of PGE_2_ generation. Together with the inhibition of mPGES-1 activity, the repression of COX-2 induction in response to inflammatory stimuli by AF3485 may contribute to the reduction of PGE_2_ biosynthesis at the inflammatory site. This off-target effects of AF3485 might be associated with enhanced antiinflammatory efficacy. Moreover, since the compound induces endothelial COX-2-dependent PGI_2_ production, both *in vitro* and *in vivo*, a protective effect on the cardiovascular system might be envisaged.

## Data Availability Statement

The datasets generated for this study are available on request to the corresponding author.

## Ethics Statement

The studies involving human participants were reviewed and approved by Ethics Committee of “G. d'Annunzio” University of Chieti-Pescara, Italy. The patients/participants provided their written informed consent to participate in this study. The animal study was reviewed and approved by National Council on Animal Care of the Italian Ministry of Health.

## Author Contributions

PP, ER, and AB conceptualized and designed the study. LF, MD, ST, and ER performed the data acquisition, analysis, or interpretation of data. PP, GF, PG-L, and AB drafted the manuscript. LF, IC, BG, GM, MA, and CM critically revised the manuscript for important intellectual content. All authors provided approval for publication of the content, and agreed to be accountable for all aspects of the work in ensuring that questions related to the accuracy or integrity of any part of the work are appropriately investigated and resolved.

## Conflict of Interest

Authors MA, BG, IC, GM and CM were employed by company Angelini Pharma S.p.A.

The remaining authors declare that the research was conducted in the absence of any commercial or financial relationships that could be construed as a potential conflict of interest.

This study received funding from Angelini Pharma S.p.A. to PP (grant number 092FM10094). The funder was involved in providing the compound AF3485 and in the performance of in vivo experiments in rats and collection of urine samples, critically revised the manuscript for important intellectual content and the decision to submit it for publication.
