# Supplementary material for: Pharmacological Characterization of the Microsomal Prostaglandin E2 Synthase-1 Inhibitor AF3485 In Vitro and In Vivo
Source: Front Pharmacol. 2020 Apr 2;11:374. doi: 10.3389/fphar.2020.00374 (PMC7147323; doi:10.3389/fphar.2020.00374)
Supplement: Supplementary file 1 [file DataSheet_1.docx]

**Supplementary Figures and Figure Legends**

**Supplementary Figure 1. Dose-response curves for inhibition LPS-stimulated human whole blood by L745337**. Increasing concentrations of L745337 (0.001-100 μg/ml) or DMSO were incubated with 1 ml of heparinized human whole blood stimulated with LPS (10 µg/ml) for 24 h. PGE_2_ and TXB_2_ were assessed by immunoassays. Results were reported as % of inhibition (mean ± SEM, n=3). The IC_50_ and the Interval of Confidence (CI) values are reported in μg/ml.

**
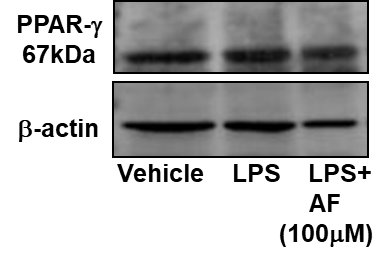
**

**Supplementary Figure 2. PPARγ expression in isolated human monocytes.** Human monocytes (1.5-2 x 10^6^ cells/ml) were incubated with vehicle or LPS (10 µg/ml) for 24 h at 37 ⁰C, in the absence and in the presence of AF3485 (100 µM); Western blot analysis of PPARγ is reported and β-actin was assessed as protein loading control.

**Supplementary Table 1. Baseline values of PGE_2_ and TXB_2_ in human whole blood and isolated human monocytes.**


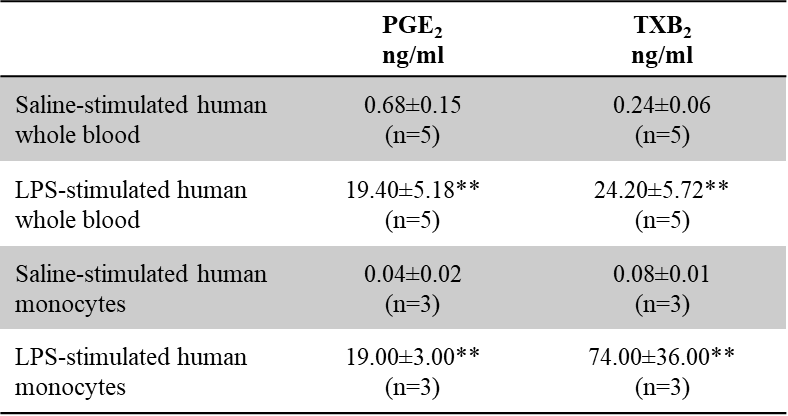


Data are reported as mean±SD; **P<0.01 versus the same prostanoid in the saline-stimulated condition.
